# Isoflurane has no effect on cognitive or behavioral performance in a mouse model of early-stage Alzheimer’s disease

**DOI:** 10.3389/fnins.2022.1033729

**Published:** 2022-10-18

**Authors:** Laura Borgstedt, Sebastian Bratke, Manfred Blobner, Christoph Pötzl, Bernhard Ulm, Bettina Jungwirth, Sebastian Schmid

**Affiliations:** ^1^Department of Anesthesiology and Intensive Care Medicine, School of Medicine, Technical University of Munich, Munich, Germany; ^2^Department of Anesthesiology and Intensive Care Medicine, Faculty of Medicine, University of Ulm, Munich, Germany

**Keywords:** Alzheimer’s disease, anesthesia, modified hole board test, transgenic mice, cognitive impairment, sex-specific differences

## Abstract

**Background:**

Patients with Alzheimer’s disease show a sex-dependent decline of cognitive and behavioral performance. It is controversially discussed whether general anesthesia itself can aggravate or even cause this neurocognitive decline. Therefore, we investigated the effect of general anesthesia on neurocognitive and behavioral function and amyloidopathy in a mouse model of early-stage Alzheimer’s disease with respect to sex.

**Methods:**

After governmental approval 10 months old Tg2576 mice and wild type (total 85 mice) either underwent general anesthesia with 1.0 minimal alveolar concentration of isoflurane for 2 h or were not exposed to isoflurane (controls). Following cognitive and behavioral testing using the modified hole board test (mHBT), brains were investigated regarding amyloidopathy, inflammation, and apoptosis. Data were analyzed using repeated measure analysis of variance (ANOVA) and univariate analysis of variance (UNIANOVA).

**Results:**

Tg2576 mice showed a decline in memory function (*p* < 0.001), less anxiety (*p* = 0.022 and *p* = 0.024), increased locomotor activity (*p* = 0.025), and impaired fine motor skills (*p* < 0.001). Amyloid precursor protein (*p* < 0.001), soluble amyloid-beta (*p* < 0.001) and insoluble amyloid deposits (*p* < 0.001) were increased in Tg2576 animals. Neither sex nor exposure to isoflurane had an effect on cognitive or behavioral testing or expression of amyloid-related biomarkers.

**Discussion and conclusion:**

We found that 10 months old Tg2576 showed typical signs of early-stage Alzheimer’s disease and corresponding histopathological alterations. Relevant sex-specific differences or an effect of isoflurane anesthesia could not be detected at this early stage of the disease.

## Introduction

Alzheimer’s disease (AD) is the most common neurodegenerative disorder worldwide. Due to medical progress and a rising life expectancy, the prevalence is increasing with an estimated total number of people living with AD dementia of 13.8 million in the U.S. in 2050 (Hebert et al., 2013). While the causative pathomechanisms are still not fully understood, the accumulation of amyloid-beta seems to play a pivotal role in the pathophysiology of AD (Querfurth and LaFerla, 2010). Other contributing factors are the accumulation of neurofibrillary bundles leading to tauopathy and the autosomal dominant genes presenilin-1, presenilin-2 (Eckel et al., 2013), and apolipoprotein E4 variant. Also, several risk factors such as age, female sex, genetic predisposition, and psychosocial factors (e.g. lifestyle, nutrition, education) have been found (Serrano-Pozo and Growdon, 2019).

According to epidemiological studies, women have a higher lifetime risk for developing AD (Seshadri et al., 1997). Neurodegeneration and clinical symptoms also occur more rapidly in females (Hebert et al., 2013), while men seem to have a shorter survival time after being diagnosed with AD (Kua et al., 2014). Whether these differences are due to a longer life expectancy, detection bias, or neurobiological vulnerability in postmenopausal females remains controversially discussed. Also, the role of an inflammatory dysregulation due to sex differences in microglia, neuroprotective effects of estradiol, or an estrogen-responsive promotor of beta-secretase (Sadleir et al., 2015) is still not fully elucidated.

While an interaction between anesthetics and amyloid-beta has been demonstrated (Eckenhoff et al., 2004), the relationship between general anesthesia and AD is still a matter of debate (Sprung et al., 2020). Since general anesthesia is usually followed by surgery, the effect of general anesthesia alone on AD patients is difficult to investigate (Lee et al., 2020). The use of animal models allows to study the effect of anesthesia on neurocognitive function without a surgical trauma.

Mice and humans are analogous regarding higher cognitive functions such as cognition, behavior and fine motor skills (Shultz et al., 2007). The Tg2576 mouse model of AD has been well characterized and is recognized to model symptoms of human AD (Schmid et al., 2019). Similar to humans, female Tg2576 develop a more severe cognitive impairment than male Tg2576 (Schmid et al., 2019).

This study aimed to investigate the effects of general anesthesia on cognitive function and behavioral parameters in a model of early-stage AD with respect to sex. We therefore decided to expose 10 months old Tg2576 to general anesthesia with isoflurane and afterward compare them to their non-transgenic littermates (both male and female) using the modified hole board test (mHBT). In order to assess amyloidopathy, neuroinflammation, and apoptosis we examined the brains for corresponding biomarkers.

## Materials and methods

This study was carried out in strict accordance with the recommendations of the Federation of European Laboratory Animal Science Associations (FELASA). The following experimental procedures on animals were approved by the Governmental Animal Care Committee (Regierung von Oberbayern, Maximilianstr. 39, 80538 Munich, Germany, Chair: Dr. B. Wirrer, Registration number: 55.2-1-54-2532-67-2016, July 28th, 2016). All surgical procedures were performed under isoflurane anesthesia, and all efforts were made to minimize suffering. Animal welfare was assessed daily.

### Mouse model

We used the B6;SJL-Tg(APPSWE)2576Kha mouse model of AD, also referred to as Tg2576. With the approval of Taconic (Taconic Europe, Lille Skensved, Denmark), male B6;SJL-Tg(APPSWE)2576Kha mice were crossed with female C57B6/SJL mice (The Jackson Laboratory, Bar Harbor, Maine, USA) in a separate breeding facility. The genotype was confirmed by PCR, using DNA from tail tissues (Charles River Laboratories, Sulzfeld, Germany). Mice homozygous for the rd1-mutation were excluded from the analysis as these mice suffer from blindness. At least 14 days prior to anesthesia and cognitive and behavioral testing, mice were transferred to a test facility for acclimatization. Mice were housed under standard laboratory conditions (specific pathogen free environment, 12 h light/12 h dark cycle, 22^°^C, 60% humidity and free access to water and standard mouse chow) (Schmid et al., 2019).

For this study, 85 mice with 10 months of age were used (median weight 28.8 g; 41 wild type (WT), 44 Tg2576; 41 male, 44 female). Mice were randomly assigned to one of eight groups (*n* = 9–12 mice per group) regarding isoflurane anesthesia or sham procedure using a computer-generated randomization list. The experimental groups were designed as depicted in Supplementary Table 1. The outcome assessor conducting the mHBT and the personnel performing the analysis of biomarkers were blinded to the group assignment.

### General anesthesia

For induction of general anesthesia mice were placed in an acrylic glass chamber that had been pre-flushed with 4.5 Vol% isoflurane (Isofluran Baxter vet, Deerfield, Illinois, USA; Vaporizer: Draeger, Luebeck, Germany) and 50% oxygen. After loss of postural reflexes, mice were placed on a warming pad (rectal temperature was measured and maintained at 37.5^°^C ± 0.5^°^C). General anesthesia was maintained for 2 h with 1.6 Vol% isoflurane (MAC 1.0) and a fraction of inspired oxygen of 50% (FiO2 0.5) administered *via* a nose chamber. Mice breathed spontaneously with an applied positive end-expiratory pressure of 5 mbar.

After 2 h mice were again placed in the acrylic glass chamber with 50% oxygen now without isoflurane until full recovery from anesthesia. Afterward the animals were weighed and placed in single cages. During general anesthesia respiratory rate, heart rate (both *via* subcutaneous electrocardiogram), gas concentrations, and rectal temperature were measured (Datex Ohmeda S/5 Anesthesia Monitor F-CM1-05 with MNESTPR Modul, Datex-Ohmeda GmbH, Duisburg, Germany).

Animals randomized to the sham procedure were placed in the same acrylic glass chamber without administration of isoflurane for 5 min, then weighed and put back into their single cages.

### Modified hole board test

The day following general anesthesia or sham procedure, respectively, mice were tested for cognitive and motor function as well as behavior using the mHBT. This test is a combination of a classical hole-board with an open field test, according to an established protocol (Gordan et al., 2012; Borgstedt et al., 2020). With this test, a total of 16 different parameters can be observed simultaneously. For the mHBT the hole-board is placed in the middle of the test arena. Ten cylinders are staggered in two lines on the board. Each cylinder contains a small piece of almond fixed underneath a grid that cannot be removed by the animals (Supplementary Figure 1). In addition, each cylinder is flavored with vanilla to attract the animals’ attention. Three of the 10 cylinders are baited with a second—approachable—piece of almond and marked with white tape. The sequence of marked holes is changed according to a protocol every day. We performed testing for 8 consecutive days. Each mouse underwent four trials per day (300 s/trial).

We evaluated two different parameters regarding behavior, cognition and motor skills, respectively. Parameters focusing on behavior are the latency with which the mice first visited the holes on the area of interest (gray board with cylinders, Supplementary Figure 1; latency to the first hole visit, LFHV) and the time spent on this board (time on board, TOB). If mice visited non-baited holes or did not visit baited holes it was summed up as wrong choice total (WCT). On the one hand, a higher total number of WCT can be interpreted as an impaired declarative memory. On the other hand, disinhibition and increased locomotor activity can lead to a reduced number of WCT as mice are e.g., less likely to omit a baited hole. Repeated choices (RC) were defined as correctly emptied holes which were revisited by the mice repeatedly. This signifies impaired working memory. An increased latency and reduced time on board represented avoidance behavior and therefore could be seen as a higher level of anxiety. The number of line crossings (LC) in the test arena served as an indicator for locomotor activity. Fine motor skills were assessed by measuring the time mice needed to eat a piece of almond which served as the bait (time for food intake, TFI). The software used to record the mice’ performance in the mHBT was The Observer^®^ 5.0 (Noldus Information Technology BV, Wageningen, Netherlands).

### Assessment of amyloidopathy, bio-markers, and reproductive stage

On day 9 after general anesthesia mice were anesthetized deeply using isoflurane and brains were harvested by decapitation using a guillotine (World Precision Instruments, Inc., Sarasota, Florida, USA). The samples were stored at −80^°^C (Thermo Scientific™ HERAfreeze™, Waltham, Massachusetts, USA). Each brain was separated into hemicortices. One hemicortex was sliced into 21 sagittal slides of 50 μm and 21 sagittal slides of 10 μm (Cryostat: Microm HM 560, ThermoFisher Scientific, Waltham, Massachusetts, USA). The other hemicortex was separated into prefrontal motor cortex, sensory cortex, and hippocampus. A total of 56 brains (7 brains per experimental group, 8 experimental groups in total) were further analyzed.

To determine the total amount of Aβ per brain (*n* = 56 brains), we homogenized brain slices of prefrontal motor cortex in 10 volumes of cold guanidine–HCl buffer (5 mol/l; 50 mmol/l Tris-Cl, pH 8.0) and mixed them for 3–4 h at room temperature. The homogenates were diluted in 10 volumes of cold casein buffer (0.25% casein; 0.05% sodium azide; 20 μg/ml aprotinin, 5 mmol/l EDTA; pH 8.0; 10 μg/ml Leupeptin in PBS), centrifuged (16,000 × g; 20 min, Biofuge fresco, Heraeus, Hanau, Germany), and the supernatant stored at −80°C (Johnson-Wood et al., 1997). The Human Aβ42 ELISA Kit with a sensitivity of 1.0 pmol/l (Human Aβ42 ELISA Kit KHB3442, RRID:SCR_008410, Invitrogen, Carlsbad, Kalifornien, USA) was used for the following ELISA. Samples and the standard solution were diluted with standard diluent buffer (Sample concentration: 0.075 μg/μl) and 100 μl of each were added to the appropriate wells and the plate was incubated in the refrigerator at 8°C on a 3D-shaker (3DL 05, Hettich Benelux B.V., Geldermalsen, The Netherlands) overnight. The next day the wells were washed four times with washing solution, 100 μl of HRP-conjugated antibody solution were dispensed into the wells and the plate was incubated at room temperature on a different 3D-shaker (Rocky^®^ 3D, Fröbel Labortechnik, Lindau, Germany) for 30 min. Wells were washed another four times with wash buffer and 100 μl Chromogen (Tetramethybenzidin) solution was added.

After then adding 100 μl of stop solution, the absorbance at 450 nm optical density was determined using the Sunrise^®^ reader (Tecan Group, Maennedorf, Switzerland) and the standard curve was generated using the Magellan^®^ software (Tecan Group, Maennedorf, Switzerland). We performed two determinations for each brain region and averaged the results for the final analysis.

To detect amyloid deposits, 50 μm thick sagittal brain slices (*n* = 21 per brain) were investigated. The slices including sensory cortex and hippocampus were fixed on microscope slides (Thermo Scientific™ SuperFrost^®^ Plus microscope slides, ThermoFisher Scientific, Waltham, Massachusetts, USA) in −20°C acetone for 20 min. The staining protocol has been described previously (Klunk et al., 2002; Burgold et al., 2011; McCarter et al., 2013). After drying at room temperature, each slice was washed twice with wash solution (PBS/Ethanol denaturized with MEK in 1:1 ratio) for 10 min. Then methoxy-X04 solution [10 mg methoxy-X04 powder (Tocris, Bioscience) diluted in 100 μl Dimethylsulfoxid, mixed with 450 μl of 1,2-Propandiol, 450 μl of PBS, and 50 μl 1 N NaOH; 800 μl of this stock was diluted with 200 ml of a 1:1-PBS/ethanol solution] was applied to the slices on a shaker in the dark for 30 min. To remove the unbonded methoxy-X04, brain slices were washed three times with wash solution and twice with distilled water for 10 min per step. In a final step, brain slices were preserved in fluorescence mounting medium (DAKO, Santa Clara, California, USA). Methoxy-X04 has a high binding affinity for amyloid deposits. The stained brain slices were imaged by magnification using fluorescence microscopy in tile scan mode (ZEISS Axio Imager, ApoTome.2 and Zen 2012 Blue Software, Oberkochen, Germany). Images were thresholded to cut off background staining. We used ImageJ software (Version 1.51 h, Wayne Rasband, National Institutes of Health, USA) to automatically measure the area covered with amyloid deposits as well as their number in the whole cortex and hippocampus.

Sensory cortex and hippocampus of seven animals per group (*n* = 56) were suspended separately in grinding tubes (Sample Grinding Kit, GE Healthcare, Munich, Germany) and 300 μl extraction-solution were added (1 ml: 970 μl Ripa Buffer; 20 μl 50 × Complete; 10 μl 100 × Phenylmethylsulfonylfluorid; 1 μl Pepstation). The samples were then homogenized using pestles (Sample Grinding Kit, GE Healthcare, Munich, Germany) for 2–3 min and afterward centrifuged for 30 min (13.000 rounds per minute at 4°C). After centrifugation the supernatant was stored at −80°C. The protein-concentration (by Bradford Assay) was standardized with Laemmli buffer [1.4 ml, 4 × times: 1 ml NuPage LDS Sample Buffer (Invitrogen NP0007); 400 μl NuPage Sample Reducing Agent (Invitrogen NP0009)]. The samples were transferred onto the gel (TGX Stain-Free™ FastCast™ Acrylamide Kit 10%; Bio-Rad Laboratories GmbH, Munich, Germany) in equal amounts (20 μl) and equal protein-concentrations (1 μg/μl) per lane for separation by gel electrophoresis and blotted onto a membrane (Amersham Hybond Low Fluorescence 0.2 μm Polyvinylidenfluorid-Membrane; TH Geyer, GmbH, Munich, Germany). The membrane was blocked for 1 h and incubated afterward with the first antibody (“APP” Cell Signaling Technology Cat# 2452, RRID:AB10694227 1:1,000, “TNF-alpha” ProSci Cat# XP-5284, RRID:AB741600 1:1,000, “Caspase 3” Cell Signaling Technology Cat# 9662, RRID:AB331439 1:1,000) overnight at 8°C on a 3D-shaker. After washing with TBS/T (1 l: 1 l dH_2_O; 3 g Tris, 11.1 g NaCl; 1 ml Tween 20) the membrane was incubated with the second antibody (“Anti-rabbit IgG” Cell Signaling Technology Cat# 7074, RRID:AB2099233 1:10,000) for 1 h on a 3D-shaker.

Following another washing step, the membrane was incubated in the dark with Clarity™ Western ECL Substrate (Bio-Rad Laboratories GmbH, Munich, Germany) for 1 min. The labeled proteins were detected with camera imaging (Bio-Rad Molecular Imager^®^ ChemiDocTM XRS +; Bio-Rad Laboratories GmbH, Feldkirchen, Germany). For analysis and normalization ImageLab^®^ was used in addition to the Stain-Free^®^ Technology to assess the total protein amount (Bio-Rad Laboratories GmbH, Feldkirchen, Germany). A standard lane was included in every blot.

Following decapitation and preparation of the brains, the reproductive cycle of each female mouse was assessed by vaginal cytology (Caligioni, 2009). Therefore, the tip of a plastic pipette (Ratiolab GmbH, Dreieich, Germany) filled with ∼10 μL saline was placed into the vagina. The vagina was flushed four times. The final flush was collected in the pipette tip and placed on a microscope slide (Thermo Scientific™ SuperFrost^®^ Plus microscope slides, ThermoFisher Scientific, Waltham, Massachusetts, USA). The material was then observed under a light microscope (Axioskop 40, Carl Zeiss Microscopy GmbH, Jena, Germany) with a 10×, 40×, and 100× objective. The full cycle in mice can be divided into 4 stages: Proestrus, estrus, metestrus, and diestrus. The determination of the respective cycle phase is based on the proportion among epithelial cells, cornified cells and leukocytes.

### Statistical analysis

Parameters obtained in the mHBT were analyzed using repeated measure analysis of variance (ANOVA) to represent the learning process over eight consecutive days as well as univariate analysis of variance (UNIANOVA) to assess the mice’ performance on day 8 of neurocognitive and behavioral testing.

Using repeated measure ANOVA, the dependent variables were tested by the between-group factors sex (female and male), genotype (Tg2576 and wild type), intervention (isoflurane anesthesia and sham), the within-group factor time (day 1–8), and their interaction terms. The values of testing on eight consecutive days are included in the model and effects of time were analyzed in a linear fashion (time) due to the strictly monotonic character of learning curves in these tests.

In calculations using UNIANOVA sex (female and male), genotype (Tg2576 and wild type) and intervention (isoflurane anesthesia and sham) served as independent variables while parameters obtained in the mHBT on day 8 served as dependent variables.

For determination of the effect size, we calculated mean differences with 95% confidence interval. *Post-hoc* tests were performed if the interaction terms sex × intervention or genotype × intervention had proven to be significant.

Regarding sample-size calculations, variables of the mHBT are considered relevant if two groups differ two times the given standard deviation. We planned a hierarchical model addressing three between-subject factors (genotype, sex, and intervention) resulting in eight randomized groups. Based on a type I error of 0.05, a type II error of 0.20 and with 64 possible *post hoc* comparisons, nine animals per group were found to be sufficient. In order to account for drop-outs we decided to aim for 12 animals per group in accordance with the Governmental Animal Care Committee.

Since distribution of the amount of amyloid-beta, the concentration of APP, Tumor necrosis factor (TNF) alpha and Caspase 3 in the western blot analysis, and as the percentage of brain area covered with amyloid deposits were positively skewed, the statistical analyses were performed following logarithmic transformation.

Results of ELISA (amyloid-beta), western blot (APP, TNF alpha, and Caspase 3) and methoxy staining (amyloid deposits) (dependent variable) were compared using UNIANOVA with the additional factors sex, genotype and intervention, and their interaction terms (independent variable).

The significance level was set at *p* < 0.05. Calculations were done with SPSS Statistics^®^ (Version 24.0; IBM; New York; United States).

## Results

### Behavioral and cognitive testing

#### Behavior

**FIGURE 1:**
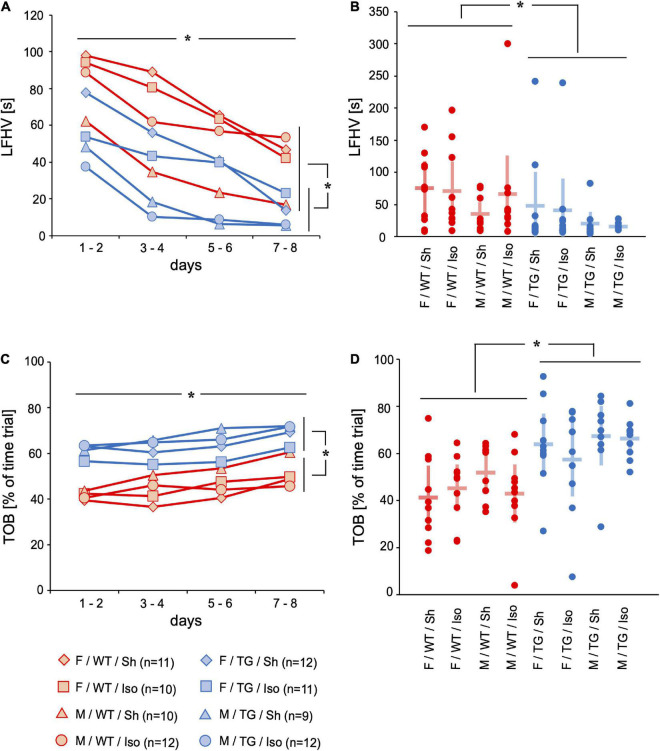
Latency to the first hole visit (LFHV) [s] and time on board (TOB) [% = fraction of time trial]. Mean of 4 trials per day on 2 days over time **(A,C)** and mean and 95%-confidence interval of 4 trials per day on all 8 consecutive days **(B,D)** sorted by group. LFHV decreased over 8 days of testing. LFHV was lower in Tg2576 compared to WT on days 7–8 and over all days. TOB increased over 8 days of testing. TOB was higher in Tg2576 compared to WT on days 7–8 and over all days. F, female; M, male; WT, wildtype (red); TG, Tg2576 (blue); Sh, sham procedure; Iso, isoflurane anesthesia. *p< 0.05.

The latency mice first visited a hole (LFHV, [s]) on the gray board of the mHBT and the time spent on this board (TOB, [% of time trial]) evaluated exploratory motivation and anxiety. Mice underwent 4 trials per day on 8 consecutive days. Each trial ended as soon as the mice found and ate the third bait or after a maximum of 300 s. LFHV decreased from day 1 to day 8 (mean difference = −56.92 s, 95%-confidence interval [−70.32; −43.54], time: *p* < 0.001). Tg2576 visited a hole earlier during the trials than WT throughout the 8 days of testing (−30.55 s, [4.61; 56.49], genotype: *p* = 0.022) and on day 8 (−30.16 s, [−51.23; −9.09], genotype day 8: *p* = 0.006; Figures 1A,B). TOB increased over 8 days of testing (mean difference = 10.90%, 95%-confidence interval [6.53; 15.27], time: *p* = 0.026). Tg2576 spent more time on board than WT over the course of the mHBT (13.12%, [1.81; 24.42], genotype: *p* = 0.024) and on day 8 (19.61%, [10.83; 28.39], genotype day 8: *p* < 0.001, Figures 1C,D).

#### Cognition

**FIGURE 2:**
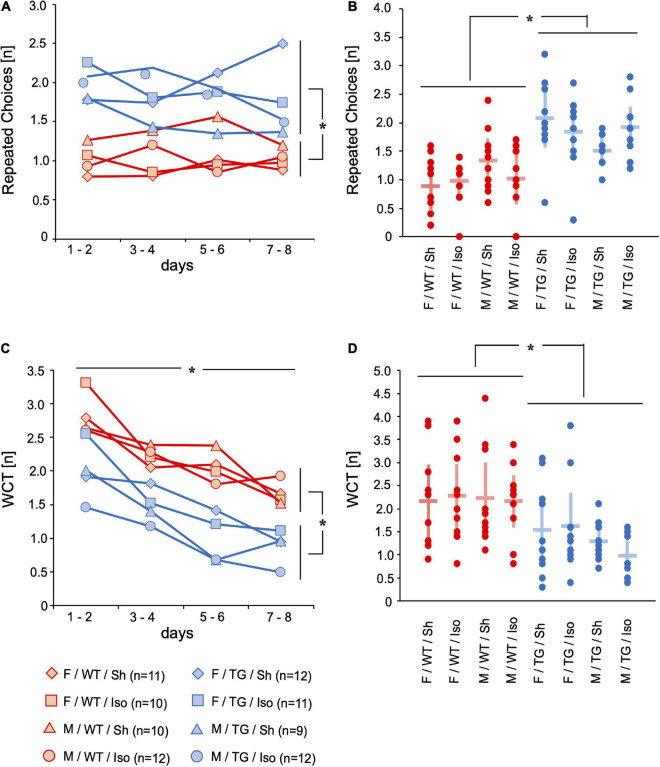
Repeated choices (RC) [n] and wrong choices total (WCT) [n]. Mean of 4 trials per day on 2 days over time **(A,C)** and mean and 95%-confidence interval of 4 trials per day on all 8 consecutive days **(B,D)** sorted by group. RC were higher in Tg2576 compared to WT on days 7–8 and over all days. WCT decreased over 8 days of testing. WCT were lower in Tg2576 compared to WT on days 7–8 and over all days. F, female; M, male; WT, wildtype (red); TG, Tg2576 (blue); Sh, sham procedure; Iso, isoflurane anesthesia. *p < 0.05.

In order to assess working memory and declarative memory, repeated choices (RC, [number per trial]) and wrong choices total (WCT, [number per trial]) were evaluated. If an animal revisited a previously emptied baited hole again, it counted as RC. If an animal did not visit a baited hole (= omission error (OE, [number per trial]) or visited a non-baited hole (= wrong choice (WC), [number per trial]) it was later summed up as wrong choices total (WCT, [number per trial]). Tg2576 did more RC than WT (mean difference = 0.81, 95%-confidence interval [0.57; 1.06], genotype: *p* < 0.001), irrespective of sex or intervention. On day 8 of the mHBT, Tg2576 visited more already emptied holes compared to WT (0.84, [0.40; 1.28], genotype day 8: *p* < 0.001; Figures 2A,B). WCT decreased over the 8 days of mHBT (mean difference = −1.40, 95%-confidence interval [−1.73; −1.08], time: *p* < 0.001). Tg2576 did less WCT compared to WT over the course of day 1 to day 8 (−0.87, [−0.46; −1.28], genotype: *p* < 0.001) as well as on day 8 (−0.61, [−0.12; −1.10], genotype day 8: *p* = 0.016; Figures 2C,D).

#### Motor skills

**FIGURE 3:**
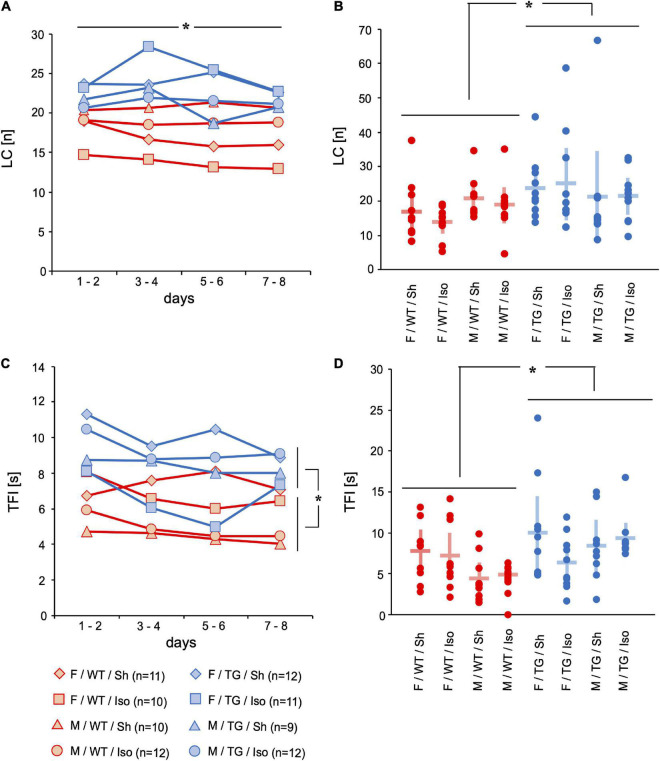
Line crossings (LC) [n] and time for food intake (TFI) [s]. Mean of 4 trials per day on 2 days over time **(A,C)** and mean and 95%-confidence interval of 4 trials per day on all 8 consecutive days **(B,D)** sorted by group. LC decreased over 8 days of testing. LC were higher in Tg2576 compared to WT over all days. TFI was higher in Tg2576 compared to WT on days 7–8 and over all days. F, female; M, male; WT, wildtype (red); TG, Tg2576 (blue); Sh, sham procedure; Iso, isoflurane anesthesia. *p < 0.05.

Motor skills were assessed with locomotor activity represented by the number of line crossings (LC, [number per trial]) during one trial and time for food intake (TFI, [s]). The floor of the four-sided mHBT apparatus is divided into 9 squares by two parallel lines between opposite walls and the gray board with the cylinders is placed in the middle (Supplementary Figure 1). Whenever an animal crossed a line, it counted as LC. Tg2576 performed more LC on the 8 consecutive days (LC, [number per trial]) than WT (mean difference = 5.2, 95%-confidence interval [0.66; 9.80], genotype: *p* = 0.025; Figures 3A,B). Tg2576 had increased TFI over 8 consecutive days than WT (mean difference = 4.7, 95%-confidence interval [2.4; 6.5], genotype: *p* < 0.001) and on day 8 (3.2, [1.4; 5.0], genotype: *p* = 0.001; Figures 3C,D).

The reproductive cycle of each female mouse was assessed (*n* = 44, WT = 21, Tg2576 = 23). Fourteen mice [31.8%, *n* (Tg2576) = 8, *n* (WT) = 6] were in estrus, 4 mice [9.1%, *n* (Tg2576) = 3, *n* (WT) = 1] in metestrus, 25 mice [56.8%, *n* (Tg2576) = 11, *n* (WT) = 14] in pro-estrus and 1 WT mouse (2.3%) in diestrus.

### Amyloid precursor protein, soluble amyloid-beta 1–42 and amyloid deposits

#### Amyloid precursor protein

**FIGURE 4:**
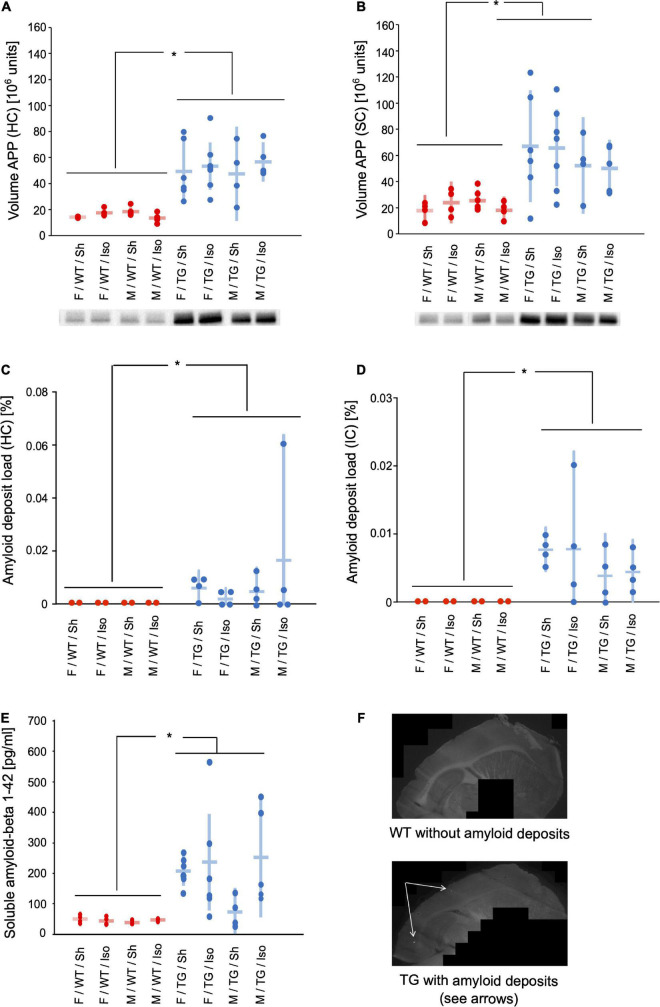
Volume of amyloid precursor protein (APP) in hippocampus (HC) **(A)** and sensory cortex **(B)** [10^6^ units] and representative western-blot bands. Amyloid deposit load in hippocampus (HC) **(C)** and isocortex (IC) **(D)** [% of total area]. Soluble amyloid beta 1–42 **(E)** [pg/ml]. Mean and 95%-confidence interval sorted by group. Representative images of methoxy staining (10 × magnification) **(F)**. APP in HC and SC, amyloid deposit load in HC and IC, and soluble amyloid beta were increased in Tg2576 compared to WT. F, female; M, male; WT, wildtype (red); TG, Tg2576 (blue); Sh, sham procedure; Iso, isoflurane anesthesia. *p < 0.05.

The expression of APP was significantly higher in both the sensory cortex (SC) and hippocampus (HC) of Tg2576 (SC: mean difference = 0.95 × 10^6^ units; 95%-confidence interval [0.60; 1.31]; *p* < 0.001; HC: 1.13 × 10^6^ units; [0.92; 1.34]; *p* < 0.001). There were no significant differences regarding sex or intervention (Figures 4A,B).

#### Insoluble amyloid deposits

The area [%] of isocortex (IC) and hippocampus (HC) covered with amyloid deposits was higher in Tg2576 (IC: mean difference = 0.0031%, 95%-confidence interval [0.0007; 0.012%]; genotype: *p* < 0.001; HC: 0.017% [0.0018; 0.16%]; genotype *p* = 0.001) than WT, where no amyloid deposits were present, irrespective of sex or intervention (Figures 4C,D,F).

#### Soluble amyloid-beta 1–42

The medial prefrontal cortex (MPFC) of Tg2576 contained significantly more soluble amyloid-beta 1–42 compared to WT (mean difference = 1.23 × 10^6^ units; 95%-confidence interval [0.91; 1.54] genotype: *p* < 0.001, Figure 4E).

### Analysis of caspase 3 and tumor necrosis factor alpha

#### Caspase 3

**FIGURE 5:**
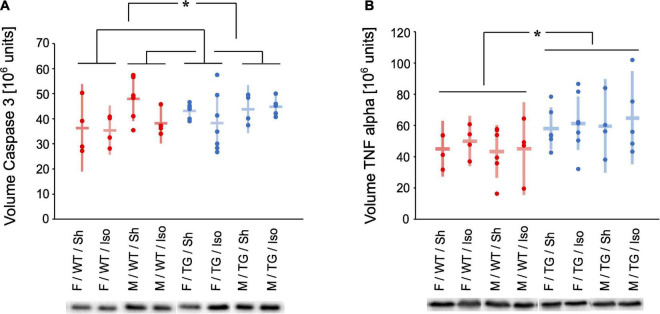
Volume of Caspase 3 **(A)** and Tumor necrosis factor (TNF) alpha **(B)** [10^6^ units] in the hippocampus and representative western-blot bands. Mean and 95%-confidence interval sorted by group. Caspase 3 was increased in male compared to female mice. TNF alpha was increased in Tg2576 compared to WT. F, female; M, male; WT, wildtype (red); TG, Tg2576 (blue); Sh, sham procedure; Iso, isoflurane anesthesia. *p < 0.05.

The expression of caspase 3 (normalized volume in 10^6^ units) in the HC was higher in male mice (Tg2576 and WT) compared to female mice (Tg2576 and WT) irrespective of genotype or intervention (mean difference = 5475613 × 10^6^ units, 95%-confidence interval [442931; 10508295], sex: *p* = 0.026, Figure 5A).

#### Tumor necrosis factor alpha

The expression of TNF alpha (normalized volume in 10^6^ units) in the HC was higher in Tg2576 compared to WT irrespective of sex or intervention (mean difference = 15365271 × 10^6^ units, 95%-confidence interval [5445146; 25285395], genotype: *p* = 0.003, Figure 5B).

## Discussion

At the age of 10 months Tg2576 mice showed a decline in working and declarative memory (more RC and less WCT compared to WT), less anxiety (reduced LFHV and increased TOB), increased locomotor activity (increased LC), and impaired fine motor skills (increased TFI). The animals had pathognomonic signs of Alzheimer’s disease in the brains like increased APP, soluble amyloid-beta, and insoluble amyloid deposits. At this early stage of the disease, we did not detect any relevant sex-specific differences nor an effect of general anesthesia.

During the course of behavioral testing all mice showed signs of habituation to the test situation (Ohl et al., 2003): increase in direct exploration (Figures 1A,B) and less avoidance behavior (Figures 1C,D). The number of repeated choices (RC) representing impaired working memory was higher in Tg2576 mice than in WT (Figures 2A,B). The number of WCT representing the declarative memory was higher in WT mice than in Tg2576 (Figures 2C,D). This might be contradictory at first glance. However, a strength of this test battery is the ability to detect interference of cognitive performance with e.g., anxiety or motivation (Ohl et al., 2001). Therefore, it is important to not only evaluate one parameter but several. WCT is the total number of wrong choices (i.e., visiting a non-baited hole, WC) and omission errors (i.e., not visiting a baited hole, OE). Since Tg2576 showed increased locomotor (Figures 3A,B) and exploratory activity (Figures 1A,B) and spent more time on the exposed part of the test arena (Figures 1C,D) than WT, they were more likely to visit any hole during the trial. These findings of enhanced movement, reduced inhibition and reduced anxiety in mice are in concordance with previous literature (Melnikova et al., 2016) and resemble symptoms in humans diagnosed with AD (Khan et al., 2020).

APP was expressed significantly more in the HC and SC in Tg2576 regardless of sex or intervention (Figures 4A,B). This resulted in an increased amyloid load in the Tg2576 mice represented by elevated amounts of soluble amyloid-beta and insoluble amyloid deposits (Figures 4C–E). The interaction between APP and amyloid-beta has been well described (Lopez Sanchez et al., 2017) and was also shown in our study. The development of AD in terms of amyloidopathy seems to be caused by an overexpression of APP (e.g., as shown in Tg2576) and an imbalance of amyloid-beta clearance (Xiang et al., 2015). Whether the amount of APP and the amyloid-beta load follow a linear correlation is still unclear and evidence suggests that these proteins also act independently (Morales et al., 2020) in AD pathology.

Caspase 3 as a marker for apoptosis was found significantly more in the HC of male mice, regardless of genotype or intervention (Figure 5A). Female mice seem to be more resilient to cytokine elevations in behavioral testing although displaying higher baseline expression of cytokine expression within the hippocampus (Hudson et al., 2014). In our study male mice although expressing higher levels of caspase 3 did not show any cognitive or behavioral alterations. This seem controversial to Hudson et al. at first glance but the animals in the aforementioned study did not receive general anesthesia, but were exposed to stress and a different mouse model was used.

TNF alpha as a sign of neuroinflammation was expressed higher in the HC in Tg2576 (Figure 5B). This finding is reflected in the results of behavioral and cognitive testing and goes in line with previous studies on AD pathology. Amyloid deposits not only co-localize with TNF alpha (Ruan et al., 2009) but are also surrounded by reactive astrocytes and activated microglia which produce cytokine among other proinflammatory molecules (Montgomery and Bowers, 2012). Also, TNF alpha immunoreactivity is increased in Tg2576 (Mehlhorn et al., 2000). Inhibition of TNF alpha has been shown to reduce monomeric amyloid-beta in a mouse model (Paouri et al., 2017) and as clinical improvement could be observed in AD patients it might also be a target for therapeutic interventions (Tobinick, 2009).

There is not only controversy whether or not general anesthesia might influence neurodegenerative diseases but also which anesthetics and what duration of exposure might be harmful or even beneficial (Hussain et al., 2014). Han et al. (2021) recently investigated the effect of short-term (30 min) vs. long-term (6 h) exposure of 5×FAD mice to isoflurane, desflurane and sevoflurane on cognitive impairment and neuropathology. They demonstrated that on the one hand short-term exposure to inhaled anesthetics did not affect hippocampus dependent memory and amyloid-beta deposition in the brain. Long-term exposure to sevoflurane and isoflurane on the other hand led to significantly increased amyloid-beta deposition in the hippocampus as well as glial cell activation in the amygdala. Although there are notable differences to our study, our findings too suggest no effect of a 2 h exposure to 1.0 MAC isoflurane on cognitive performance and AD pathology.

The lack of sex-differences and the missing effect of isoflurane might be explained by our study conception. In a previous study from our department, female Tg2576 showed a pronounced cognitive decline at the age of 12 months when compared to male Tg2576 at 12 months. A female sex-dependent correlation of cognitive impairment to the amount of soluble amyloid-beta and amyloid deposits has also been demonstrated (Schmid et al., 2019). We therefore decided to expose Tg2576 of 10 months to general anesthesia in this follow-up study as we expected a potential sex-dependent effect of isoflurane. To avoid a possible floor effect, we specifically refrained from performing the experiments on older animals in which a sex difference was already apparent.

Besides the missing effect of our intervention our study has other limitations. We chose to use an AD mouse model restricted to amyloidopathy, although in humans living with AD both amyloidopathy and tauopathy are found (Lane et al., 2018). Recent studies suggest that the amyloid cascade hypothesis alone may not fully capture the complexity of AD (Caselli et al., 2020) proposing a link between amyloid-beta deposition and intraneuronal neurofibrillary tangle formation (He et al., 2018) and addressing the importance of amyloid-beta clearance (Frisoni et al., 2022). Also, the role of non-proteinaceous pathologies such as activated microglia, dystrophic or p-tau-positive neurites, and reactive astrocytes around plaques as well as granulovacuolar degeneration, cerebral amyloid angiopathy, and blood-brain barrier dysfunction are discussed (Husain et al., 2021). Although our study fails to investigate the neuronal microenvironment and non-proteinaceous pathologies, insights into the complex interplay between general anesthesia, neuroinflammation, and clinical and histopathological signs of AD are given. As a limitation the female mice were not synchronized according to their estrous cycle. At the time of decapitation 88.6% of the female mice in this study were in pro-estrus (56.8%) or estrus (31.8%) which are the stages of the mouse reproductive cycle with the highest levels of estrogen. These high levels of estrogen might have served as a neuroprotective factor (Gurvich et al., 2020) but as we did not detect any sex differences the effect might be minimal. Another limitation is that biomarkers and the amyloid cascade have only been assessed at one time point. In order to keep the number of animals and their stress levels as a factor of interference with the mHBT as low as possible we decided to use this approach and avoided taking blood samples on a regular basis.

## Conclusion

Tg2576 mice show typical signs of early-stage Alzheimer’s disease at 10 months of age: decline in working and declarative memory, less anxiety, increased locomotor activity, and impaired fine motor skills. We also found histopathological alterations in the brains typical for Alzheimer’s disease. Sex or general anesthesia do not seem to have an effect on cognition, behavior, or motor skills at this early stage of the disease.

## Data availability statement

The raw data supporting the conclusions of this article will be made available by the authors, without undue reservation.

## Ethics statement

The animal study was reviewed and approved by Regierung von Oberbayern, Maximilianstr. 39, 80538 Munich, Germany, Chair: Dr. B. Wirrer, Registration number: 55.2-1-54-2532-67-2016, July 28th, 2016.

## Author contributions

LB: investigation, formal analysis, validation, writing – original draft and review, and editing. SB: investigation, writing – review, and editing. MB: methodology, resources, formal analysis, supervision, writing – review, and editing. CP: investigation and formal analysis. BU: formal analysis and validation. BJ: conceptualization, methodology, formal analysis, validation, supervision, writing – review, and editing. SS: conceptualization, methodology, investigation, formal analysis, validation, data curation, writing – original draft and review, and editing. All authors contributed to the article and approved the submitted version.
