# Supplementary material for: Isoflurane has no effect on cognitive or behavioral performance in a mouse model of early-stage Alzheimer’s disease
Source: Front Neurosci. 2022 Oct 18;16:1033729. doi: 10.3389/fnins.2022.1033729 (PMC9622753; doi:10.3389/fnins.2022.1033729)
Supplement: Supplementary file 1 [file Data_Sheet_1.PDF]

frontiers

Supplementary Material

Supplementary Table 1: experimental group design

Age (months)	Sex	Genotype	Intervention	n
10	Female	WT	Sham	11
10	Female	WT	Isofluran	10
10	Male	WT	Sham	10
10	Male	WT	Isofluran	10
10	Female	Tg2576	Sham	12
10	Female	Tg2576	Isofluran	11
10	Male	Tg2576	Sham	9
10	Male	Tg2576	Isofluran	12
