# Supplementary figures and images for: Isoflurane has no effect on cognitive or behavioral performance in a mouse model of early-stage Alzheimer’s disease
Source: Front Neurosci. 2022 Oct 18;16:1033729. doi: 10.3389/fnins.2022.1033729 (PMC9622753; doi:10.3389/fnins.2022.1033729)

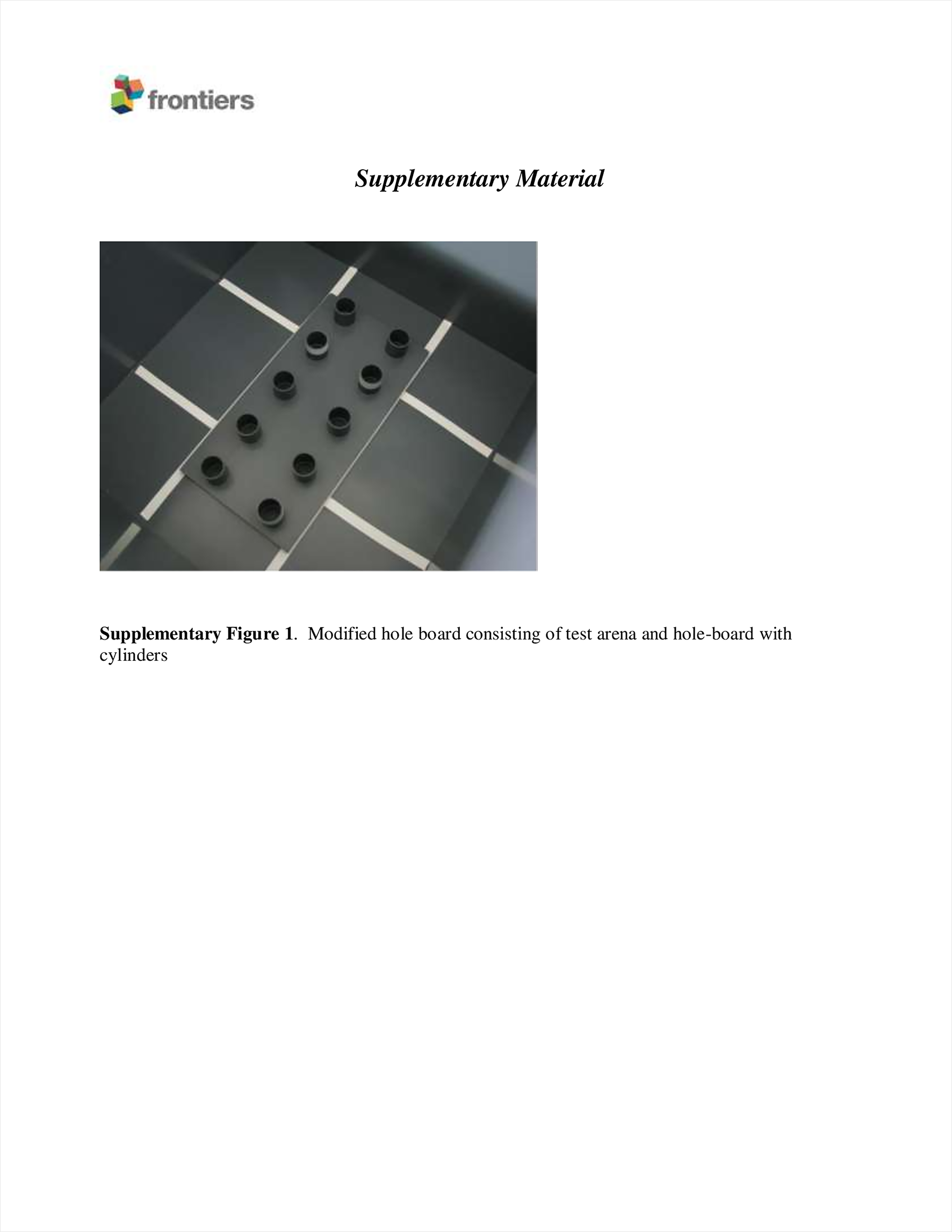

Supplement: Supplementary file 2 [file Image_1.TIFF]
